# Forest Environmental Carrying Capacity Based on Deep Learning

**DOI:** 10.1155/2022/7547645

**Published:** 2022-09-27

**Authors:** Song Linshu, Wang Hao, Yang Chao, Song Weiming, Wang Siyi, Wang Shen

**Affiliations:** ^1^College of Economics and Management, Beijing Forestry University, Beijing 100083, China; ^2^Chinese Research Academy of Environmental Sciences, Beijing 100012, China

## Abstract

In this paper, we proposed an assessment system of forest environmental carrying capacity from many aspects and comprehensively evaluated and predicted the forest environmental carrying capacity of 40 cities in the Yangtze River Delta of China by using the proposed deep learning-based model. In addition, the proposed model is used to dynamically evaluate the comprehensive scores of forest environmental carrying capacity of 34 provinces and cities in China between 2015 and 2020. This paper also has the following contributions: (1) a deeply integrated unidirectional bidirectional LSTM considering the correlation of time series is proposed. The bidirectional LSTM network is used to deal with the backward dependence of input data to prevent the omission of some useful information, which is beneficial to the prediction results. (2) In terms of missing data processing, the Mask layer is added to subtly skip the processing of missing data, which will help to enhance the evaluation results.

## 1. Background

The decline of ecological environment quality directly threatens the quality of life and health of human beings [1, 2]. The term “health,” which was first used in medicine to describe the life state and behavior of organic individuals, was adopted and developed by ecologists and environmentalists in their respective fields. For example, several concepts have been proposed by some scholars: environmental health, environmental medicine, forest ecosystem health, wetland ecosystem health, watershed health, landscape health, etc. [3]. Forest ecosystem health has been accepted and used more and more by ecologists, forestry, and natural resource management scholars, and “forest health” has been regarded as the standard and goal of forest status evaluation and forest resource management, and the assessment of forest ecosystem health has been put forward. After the 1990s, a series of monitoring programs have been initiated around the world [4] such as EMAP (environmental monitoring and assessment program) and FHM (forest health monitoring). It is used to understand forest state and assess forest ecosystem health, opening up a research approach to explore sustainable research, development, and utilization of forest resources.

Ecological carrying capacity is an important basis for evaluating regional sustainable development, as well as an important indicator to measure the degree of human social and economic activities on the utilization and interference of natural resources [5]. As a component of the terrestrial ecosystem with the largest area and highest biomass, forest carrying capacity will directly affect global ecosystem security and human survival and development [6]. Therefore, the study of forest ecological carrying capacity is of great significance for coordinating the relationship between regional forest resources and sustainable economic and social development.

Since American scholars, Parker and Burgess, first defined carrying capacity as the maximum number of an individual living creature in a specific environment in 1921 [7], many foreign scholars have discussed the connotation and research methods of carrying capacity. Meadows from MIT constructed the famous “World Model” [8] by using the system dynamics model in The Limits to Growth, making carrying capacity research the core hot issue in this field for the first time. Then, Slesser began to apply the ECCO model to the system dynamics model [9]. Daily and Ehlrich [10] focused on the relationship between population and Earth carrying capacity. Saveriades [11] proposed the concept of tourism carrying capacity for the first time in their study of ecological carrying capacity on the east coast of Cyprus.

Zhang et al. [12] believed that carrying capacity theory is a philosophical issue related to the future fate of mankind and will always be a hot and difficult issue in ecological research. Liu and Zhu [13] discussed the comprehensive, regional, and human-land relationship of geography in the application of resource and environmental carrying capacity. By definition, Gu [14] thinks that ecological carrying capacity is to point to in a certain time, within the scope of a certain space, the ecological system under the action of self-adjusting and positive human healthy and orderly development, and the ecological system can support the resource consumption and environmental pollution degree, the intensity of social and economic development, and the population of a certain level of consumption. Huang and Ren [15] studied the relationship between ecological carrying capacity and ecological security. In terms of research content, Yang and Yang [16] and Fu et al. [17] have evaluated the natural resources and environmental carrying capacity of Gansu province and Hainan Province from the provincial perspective, respectively. Jiao et al. [18], and Yu and Sun [19] studied water ecological carrying capacity and land ecological carrying capacity, respectively, from the perspective of resource types. In terms of research methods, Li et al. [20] conducted a comprehensive evaluation of Marine ecological carrying capacity in Shandong Province by using the artificial neural network method. Ma and Wang [21] used system dynamics to simulate and predict the ecological carrying capacity of Dalian city. Tian et al. [22] studied the ecological carrying capacity of Hubei Province by using the ecological footprint model.

## 2. Index System of Forest Ecosystem Health Assessment

Forest ecosystem health evaluation is mainly based on the stability and sustainability of forest ecosystem and the completeness (integration) of ecosystem structure and function. The stability, sustainability, and integration of ecosystem are the foundation of ecosystem health and the standard of forest ecosystem health evaluation. Only when the structure of an ecosystem is complete and the system is relatively stable, can it fully realize its ecological process and ecological function, and maintain the sustainability of the system, such an ecosystem is a healthy ecosystem. Evaluation factors include ecosystem vitality, organizational structure, resistance, and resilience.

Vigor refers to the energy input and nutrient cycling capacity of ecosystem, and its specific indicators are primary productivity and material cycling of ecosystem. Resilience refers to a system's ability to overcome stress and bounce back when the stress is gone. Organization refers to the complexity of the system, which will change and function with the secondary succession of the ecosystem.

The forest vigor can be measured by net primary productivity (NPP) of forests, biomass, and metabolism. NPP is mainly measured by experiment and investigation, biomass is mainly calculated by investigation and model, and metabolism can be measured by biological methods.

Organizational structure refers to the species composition structure of the system and the relationship between species, reflecting the complexity of ecosystem structure. The organizational structure of ecosystem includes two meanings: one is the species diversity of ecosystem, and the other is the complexity of ecosystem. The meaning of species diversity includes both the number of existing species and the relative abundance of species.

Forest adaptation includes resilience, which refers to the system's ability to gradually recover in the absence of external stress, and resistance, which refers to the system's ability to resist external disturbance. It is difficult to measure resilience and resistance directly.

The resistance and resilience of forests are generally measured by indirect means. In forest health evaluation, the degree of forest pests and diseases in the study area or forest fire risk level can be selected to measure.

## 3. Determination of Biomass

Table 1 shows the evaluation index system in this article, and the following section in section 3 will introduce those indicators for details.

### 3.1. Sample Wood Standard

The standard tree in the ground for each wood inspection chooses the most close to the average diameter of the DBH 1 tree as a sample wood, and the selection of sample wood should avoid the following conditions: trunk serious bending, trunk section serious deformation, double branches, no roof, disease rot, the main shoot, or large branch dead.

### 3.2. Determination of Tree Layer Biomass

On the basis of standard field investigation, the relative growth method is adopted to calculate the biomass of tree layer. The relative growth method is the use of DBH square by tree height (*D*^2^*H*) as the independent variable of fitting model, it can more truly reflect the biomass with diameter at breast height (*D*) and the change trend of height (*H*), and the calculating formula is *W*=*a*(*D*^2^*H*)^*b*^ or *LnW*=*Lna*+*bLn*(*D*^2^*H*), so through the survey of standards of *D* and *H*, we can work out standard ground biomass, and then calculating the biomass per hectare.

In standard plots, DBH (*D*) and height (*H*) of tree species were measured and recorded. The selected sample wood should be measured tree height, DBH, and crown; marked at 1.3 m on the ground; and marked on the trunk of the north and south orientation. After finishing the retest, we cut down the sample wood and measure the height of the live branch and the diameter of the trunk at that height immediately. We measure the height and azimuth of branches one by one from bottom to top and number them one by one. All branches were sawed together to separate branches and trunks, and then, the biomass of trunks, branches, leaves, and roots was measured, respectively.

(1)   Biomass of trunk: after removing the trunk of the branch, the disc was cut in sections from the trunk base to the top tip, with specific heights of 0 m, 1 m, 1.3 m, 3 m, 5 m, and 7 m. The last tip of the old tip should be reserved and weighed. The sample tree number, the disk number, the height of the section, and the north-south orientation were marked on the nonworking surface. Then, the diameters of each ring on the disk section were measured in the north-south and east-west vertical directions according to the requirements of the tree science. The volume of wood and bark in each section was calculated according to the skin diameter, peeling diameter, and dividing section length. Then, the bark and wood were separated, and their volumes were measured, respectively. After being dried in an oven at 85°C for 48 h, the bark was taken out and weighed to calculate the volume weight of wood and bark. Then, according to the volume of wood and bark in the segment and their volume weight, the dry matter weight of wood and bark in the segment was obtained. The total dry matter weight of wood and bark per tree was obtained by accumulative values of each section of the same sample tree.
(2) Biomass of branch: base diameter (*D*_0_) and branch length (*L*) of all branches of the whole tree were measured branch by branch. Ten branches with different sizes were selected as samples, and the samples were marked. The length of the segment was determined according to the diameter and length of branches. Cut branches in each segment to distinguish the segment of the disc. The dry matter weight of wood and bark of sample branches was measured and calculated by the method of trunk. Add the wood and bark together to get the total weight of dry matter (*W*). Taking *D*_0_^2^*L* of the sample branch as independent variable and dry matter weight (*W*) of the branch as dependent variable, the regression analysis was conducted according to *W*=*a*(*D*_0_^2^*L*)*b*, and parameters *a* and *b* were obtained to establish the regression equation of the branch biomass. The *D*_0_ and *L* of all branches of a single sample tree were substituted into the regression equation one by one to calculate the biomass of all single branches, and the biomass of sample wood was obtained by summing them up.
(3)   Determination of leaf biomass: the sampled branches were selected to pick the leaves and weigh the fresh weight, and a small number of leaves were selected as samples. The fresh weight of the extracted leaf samples should be immediately weighed, and the leaf samples should be put into a drying oven immediately after being brought back indoors, and dried at a constant temperature of 85°C for 24 h. The dry matter ratio of leaves was calculated, and the dry matter weight of leaves per branch was further calculated. Then, with the dry matter weight of leaf as the dependent variable, the regression equation between the dry matter weight of leaf and *D*_0_^2^*L* was established by using the same method as the biomass of branches, and the dry matter weight of the whole plant was calculated by the relative growth method.
(4) Biomass of root: dig out all the root piles and roots of the sample wood as far as possible, and classify them according to their coarseness. Weigh the fresh children of root piles and roots of each coarseness and take samples to weigh their fresh weight. After drying for 48 h, the drying weight of the sample was weighed, and the dry matter ratio of each component and the dry matter weight of each root were calculated. We total the dry matter weight of root pile and root of all levels, namely, root biomass of the sample wood.

#### 3.2.1. Determination of Biomass of Shrub Layer

Two to five representative quadrats were selected to dig out all shrubs with roots, separate branches, and roots, and weigh the fresh weight of stems, branches, leaves, and roots by species. Meanwhile, the wet weight and drying weight of different parts of each species were sampled to calculate the dry matter rate, and the biomass of each species and the total biomass of shrub layer in the quadrat were further calculated, and the total biomass of shrub layer per hectare was converted.

#### 3.2.2. Determination of Biomass of Herbaceous Layer

Two to five representative quadrants were selected, and all herbaceous plants were cut down to the ground and weighed for fresh weight. A 0.5 m × 0.5 m quadrant was set in one corner of the quadrant, and the roots of all herbaceous plants in the quadrant were dug out and weighed for fresh weight after the soil was removed. At the same time of weighing fresh weight, the aboveground part and root system were, respectively, weighed. After drying, the drying weight was weighed, and dry matter rate was calculated. Finally, biomass and biomass per hectare were calculated.

#### 3.2.3. Calculation of Total Biomass

The biomass of the whole community can be obtained by summarizing the biomass of each layer of tree, irrigation, and grass per unit area, and then, the subfunction relationship between the total biomass and DBH (*D*) and height (*H*) can be established by using formula *W*=*a*(*D*^2^*L*)*b*. In this way, the biomass of the sample plot can be calculated by investigating *D* and *H* of the sample plot, and then, the biomass of each hectare can be calculated.

### 3.3. Determination of Plant Diversity Index

The following factors should be considered in the selection of forest plant diversity indicators:

1. The selected plant diversity index is simple and easy to operate.
2. The selected index must be a standard quantity at a certain spatial scale, which should not only quantitatively represent the relative size of plant diversity in a specific community but also be comparable in analyzing the relative size of plant diversity among forest communities.
3. The selected plant diversity index should reflect the differentiation of plant diversity of selected community species and each structural community as much as possible, and reflect the spatial differentiation characteristics of plant diversity quantity in the community to a certain extent.
4. The characteristics of specific community data should be considered, which should not only meet the variable requirements in the diversity index formula but also have clear ecological significance.

According to the above principles, research needs, and collected data, the Simpson diversity index was selected in this study to represent the forest plant diversity. Simpson diversity index, as an analysis index, reflects the plant diversity of the community. This diversity index can basically be employed to handle the spatial distribution and change characteristics of the plant diversity of the forest community, and its calculation formula is as follows:

(1) $$\begin{array}{c} S=\frac{N\left(N-1\right)}{\overset{m}{\underset{i=1}{\sum }}n_{i}\left(n_{i}-1\right)}\text{,} \end{array}$$

where *S* is Simpson diversity index of the overall species in the forest community, *N* is the total number of individuals, *n*_*i*_ is the number of individuals of the ith species, and *m* is the number of species.

### 3.4. Classification of Forest Fire Risk Levels

Forest fire is the natural enemy of forest, and it is a natural disaster with wide occurrence, great harm, strong timeliness, and difficult disposal and rescue in the world. The basic principle of forest fire risk classification is based on the “National Forest Fire Classification” as the standard, but it is different from the national classification. For forest fire danger division using clustering method, the influence of many factors, forest fire selection effect on fire bigger height, crown density and management measures, the distance from the distance from the tourist spots, advantage tree species, forest-road six factors as factor analysis, clustering to area, and 40 small class as a unit, the cluster factors of each cluster unit were investigated and assigned according to the actual survey data. Then, according to the actual experience, the SPSS statistical software was used for cluster analysis, and the threshold value was 0.8. The Forest Farm was divided into three fire risk levels:

*Level I Danger Zone*. The distribution area is characterized by large population density, large population mobility, close distance to the forest road, convenient transportation, railway line, and road through different site conditions of forest types of combustible materials accounted for a large proportion, which are the area of high fire risk, in the forest health evaluation value of 0.4.

*Level II Danger Zone*. The distribution area is characterized by close proximity to tourist spots and roads, and its stand also has obvious characteristics. The stand structure is complex, with more shrubs and dead objects in the forest, and the proportion of combustible objects in different site types is large. The value of this area in forest health evaluation is 0.4.

*Level III Danger Zone*. Located in the southwest corner of Forest Farm, the distribution area is characterized by relatively concentrated small classes, far from tourist spots, mostly natural broadleaved mixed forest, steep slope, high altitude, and little human interference. The value of this area in forest health evaluation is 0.8.

## 4. The Comprehensive Evaluation Model Construction for Forest Carrying Capacity

### 4.1. Recurrent Neural Network

Cyclic neural network can well process sequence information and learn effective features from it, and is applied to image [23], speech recognition, and other fields in the early stage [24]. In recent years, RNN has also achieved good performance on tasks in the field of NLP. Lin et al. [25] used hierarchical RNN to model documents and proposed a two-step training method, which achieved good results in sentence ordering task and oral translation task. Liu et al. [26] proposed a multi-time-scale LSTM model extracts sentence and document representation. Experimental results on four data sets indicate that this model is superior to other neural network models. Zhou et al. [27] constructed a bidirectional LSTM network based on an attention mechanism to learn features from sentences for relational classification, only considering the word vector of sentences, without considering other lexical or syntactic features generated by NLP tools. In document-level emotion classification tasks, researchers generally construct hierarchical neural network models to learn document feature representation. For example, Tang et al. [28] first used the LSTM network to learn sentence vector representation from word vector and then used bidirectional threshold RNN (gated RNN) to learn document vector representation from sentence vector. Chen et al. [29] constructed a two-layer LSTM network and considered the attention mechanism of users and products.

RNN can not only learn the information in the sentence but also learn useful feature representation from the syntax tree path. Roth and Lapata [30] used the LSTM network to extract features of dependency paths for semantic role labeling. In terms of the task of relation classification, Xu et al. [31] adopted multichannel LSTM network and considered four dependency paths of words, parts of speech, dependency relation, and hypernym among entities. Cai et al. [32] proposed a RNN network, which contains bidirectional two-channel LSTM network, learning word path, and dependency path are two characteristic representations of dependency path. Similarly, the proposed model includes a bidirectional LSTM network for learning valid features from syntactic paths.

### 4.2. LSTM Network Structure

**Figure 1:**
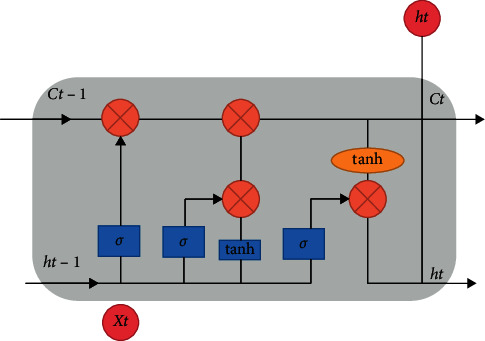
The architecture of LSTM.

The LSTM network structure was first proposed by Hochreiter and Schmidhuber in 1997 [33].  Figure 1 shows the structure diagram of an LSTM basic unit. Like RNN, LSTM elements have layer input *x*_*t*_ and layer output *h*_*t*_ at each iteration. The complex unit also takes into account the unit input state $\tilde{C_{t}}$, the unit output state *C*_*t*_, and the previous unit output state *C*_*t*−1_ when training and updating parameters.

**Figure 2:**
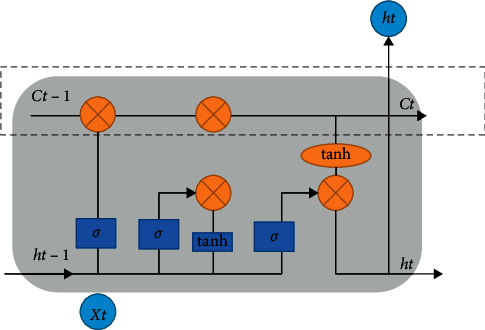
Constant error transmission structure.

In LSTM network, the basic structure of the hidden layer is a storage unit, and the structure shown in Figure 2 is an LSTM cell structure unit. It contains one or more memory cells and a pair of adaptive, multiplicative connectivity cells that connect inputs and outputs to all cells in the block. At the core of each memory cell, it is a circular self-connecting linear cell, commonly known as “constant error transmission,” and the activation of this cell is called “cell state,” which is the key to LSTM. The constant error transmission of a unit is shown in the dotted box in Figure 2.

**Figure 3:**
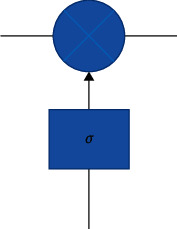
Gate control structure.

Constant error transmission solves the vanishing error problem: in the absence of new input or error signals, the local error return of constant error transmission remains unchanged, neither growing nor decaying. However, if only constant error conveyor belt, LSTM structure is unable to realize the addition and deletion of information flow. Therefore, a similar gating structure is proposed to realize the screening in the process of information transmission. As shown in Figure 3, the gated structure cleverly filters the information, mainly through a neural layer of sigmoid function and a point-by-point multiplication operation. Through the sigmoid layer structure, an output value between 0 and 1 can be obtained. These output values imply a weight assignment of passing information (e.g., 0 means that no information is allowed to pass through the structure, and 1 means that all input information can pass through the structure).

In summary, LSTM unit includes 3-gate structures. These gated structures, especially forgetting gates, help LSTM become an efficient and extensible model for solving several learning problems related to sequential data. For the *t* th time step, the forgetting gate, input gate, and output gate are expressed as *f*_*t*_, *i*_*t*_, and *o*_*t*_, respectively.

The first step we need to consider in the LSTM network unit is what redundant information the network structure wants to discard that we do not care about. This decision requires the forgetting gate structure to operate. The forgetting gate will receive the input *x*_*t*_ of this unit and the output *h*_*t*−1_ of the hidden layer of the previous time step as the input of the cell unit at this moment, and learn according to the following formula (2). It learns a real value between 0 and 1 for each item in *C*_*t*−1_, so as to control the degree of forgetting of the state of the previous unit:

(2) $$\begin{array}{c} f_{t}=\sigma_{g}\left(W_{f}x_{t}+U_{f}h_{t-1}+b_{f}\right)\text{,} \end{array}$$

where *W*_*f*_ is the weight matrix that maps the hidden layer input to the forgetting gate, *U*_*f*_ is the weight matrix that connects the output state of the previous unit to the forgetting gate, and *b*_*f*_ is the bias vector. *σ*_*g*_ is the gate activation function. Usually, the hyperbolic tangent function (tanh) is used as the activation function in LSTM structures.

The next step is to decide how much new information to add to the cellular state. An input gate and a hyperbolic tangent function (tanh) control what new information is added. Then, according to the output *f*_*t*_ of the forgetting gate obtained previously, it is used to control the extent to which the information of the previous unit is forgotten, and the output *i*_*t*_ of the input gate is used to control how much new information is used. Then, we can update the unit state of the memory unit according to the following formulas:

(3) $$\begin{array}{c} i_{t}=\sigma_{g}\left(W_{i}x_{t}+U_{i}h_{t-1}+b_{i}\right)\text{,} \end{array}$$

(4) $$\begin{array}{c} \tilde{C_{t}}=\tanh\left(W_{C}x_{t}+U_{C}h_{t-1}+b_{C}\right)\text{.} \end{array}$$

Similarly, *W*_*i*_ and *W*_*C*_ are weight matrices that map hidden layer input to legacy input gate and input unit state, respectively, while *U*_*i*_ and *U*_*C*_ are weight matrices that connect previous unit output state to input gate and input unit state, respectively. *b*_*i*_ and *b*_*C*_ are bias vectors.

Finally, we need to make sure that our network produces the desired output, which will be based on our cell state. The output gate is employed to consider how much information the network needs for further learning. Similar to the updating of the two parts of the input gate, the final output content of the output gate is also determined by activation function, as shown in the formula (4). The tanh function is then used to process the content of cell state (formula (5)). Each computed Ct value may not be within the range of −1 to 1, so it needs to be adjusted. We multiply these two pieces and get the part that we want to output.

(5) $$\begin{array}{c} o_{t}=\sigma_{g}\left(W_{o}x_{t}+U_{o}h_{t-1}+b_{o}\right)\text{,} \end{array}$$

(6) $$\begin{array}{c} h_{t}=o_{t}*\;\tanh\;\left(C_{t}\right)\text{.} \end{array}$$

Similarly, *W*_*o*_ is the weight matrix that maps the hidden layer input to the output gate, while *U*_*o*_ is the weight matrix that connects the output state of the previous unit to the output gate. *b*_*o*_ is the bias vector. According to the results of the four equations above, during each iteration, unit output state *C*_*t*_ and layer output *h*_*t*_ can be calculated as follows:

(7) $$\begin{array}{c} C_{t}=f_{t}*C_{t-1}+i_{t}*\tilde{C_{t}}\text{.} \end{array}$$

Finally, the *C*_*t*_ obtained is the cell state of the hidden layer unit.

The final output of the LSTM layer should be a vector of all outputs, represented by *Y*_*T*_=[*h*_*T*−*n*_, ⋯, *h*_*T*−1_].

### 4.3. Two-Way LSTM Network Prediction Model

Bidirectional propagation LSTM network (Bi-LSTM) is also a deformation of bidirectional cyclic network structure. So, let us first introduce bidirectional cyclic networks (BiRNN).

#### 4.3.1. BiRNN Network

Since the classical RNN usually include the time step lag among the input and the target when handling the sequence problem, some context information can be given to the network; that is, the future information of *T* step time step can be added to obtain the output together. Theoretically, *T* can be very large to capture all future available information, but in fact, it is found that if *T* is too large, the accuracy of prediction results may decline. This is because the network concentrates on learning and memorizing a large amount of input information and does not reasonably learn the input information, which leads to the decline of the joint modeling ability of the prediction model.

BiRNN can be modeled by processing forward and backward data propagation. Such bidirectional transmission not only provides historical knowledge for each neuron node in the network but also imperceptibly learns the trend of subsequent changes.

In the forward propagation layer, a forward hidden layer output *h*_*t*_^*f*^ is obtained through a nonlinear transformation of input data according to the formula (8). In the backward propagation layer, a nonlinear transformation is performed on the input data according to the formula (8) to obtain a backward hidden layer output *h*_*t*_^*b*^.

(8) $$\begin{array}{c} h_{t}^{f}=\sigma \left(W_{hh}^{f}h_{t-1}^{f}+W_{xh}^{f}x_{t}+b_{f}\right)\text{,} \end{array}$$

(9) $$\begin{array}{c} h_{t}^{b}=\sigma \left(W_{hh}^{b}h_{t+1}^{b}+W_{xh}^{b}x_{t}+b_{b}\right)\text{.} \end{array}$$

Two recurrent neural network (RNN) hidden layers are trained, and the two independent hidden layers pass through a connection layer. According to the formula (10), the output of forward transfer layer and the output of reverse transfer layer are combined into the same output layer.

(10) $$\begin{array}{c} h_{t}=W^{f}h_{t}^{f}+W^{b}h_{t}^{b}+b_{h}\text{.} \end{array}$$

### 4.4. Structural Optimization of the Prediction Model

For the network framework we have built, we need to refine our predictions. Predecessors have tried a variety of methods to optimize the model as a whole. In our article, the dropout and early-stop algorithms are employed to optimize the proposed framework to promote the Acc of the proposed method.

### 4.5. Dropout Principle

Hinton proposed a method called dropout, which can effectively reduce the occurrence of overfitting, which is equivalent to adding a regularization constraint to the model. This method will be employed in this paper.

## 5. Experiment and Result Obtained

In order to unify standards and ensure the reliability of experimental results, it is generally necessary to preprocess the original data before modeling, which is commonly referred to as standardized data processing. We need to transform the original data into dimensionless standardized values to eliminate the influence caused by different attributes among different indicators, so as to make the results more comparable. Otherwise, the accuracy of experimental results may be affected to some extent.

(11) $$\begin{array}{c} X^{\prime }=\frac{X-X_{\max}}{X_{\max}-X_{\min}}\text{,} \end{array}$$

(12) $$\begin{array}{c} X=2*X^{\prime }-1\text{.} \end{array}$$

*X*_max_ is the maximum value in the sample instance, and *X*_min_ is the minimum value in the sample instance. The normalized sample data *X*′ are scaled to a value between −1 and 1 through the formula (12).

### 5.1. Standardization of Factors

As the evaluation factors of the evaluation index system come from different aspects, it is difficult to directly evaluate them, because the dimensionality between the coefficients is not unified, so there is no comparison. Even for the same parameters, although their impact on health can be judged according to the size of their measured values, there is still a lack of a comparable environmental standard to accurately reflect their contribution to health. Therefore, the evaluation factors must be quantified and the incomparability of parameters can be solved by standardized methods. The standardization method is as follows:

(13) $$\begin{array}{c} X_{j}=\frac{X_{i}}{Y_{\max}}\text{,} \end{array}$$

where *X*_*j*_ is standardized index value; *X*_*i*_ is the value of the selected index; and *Y*_max_ the highest value in a class of indexes.

### 5.2. Forest Ecosystem Health Result

#### 5.2.1. Classification of Forest Health Status

In the process of forest health evaluation, this paper chooses BP neural network model to evaluate the forest farm. According to its evaluation method and selected index system, on the basis of systematic analysis and integration of existing research results at home and abroad, the forest ecosystem health evaluation method based on Bi-LSTM is constructed. The grading standards of health are indicated in Table 2.

#### 5.2.2. Prediction of Forest Carrying Capacity

This paper takes 40 urban forests in the Yangtze River Delta of China as research samples.  Table 3 shows the forest health condition of the cities.

As can be seen from Table 3, there are 7 subhealth areas in carrying capacity, accounting for 17% of the total number of major areas. There are 18 districts, or 45 per cent of the main districts, with average health. There were 5 undesirable areas, accounting for 12.5% of the total number of major areas. Only two districts, or 5 percent of the total, are healthy.

#### 5.2.3. Forest Carrying Capacity in Yangtze River Delta from 2015 to 2020

**Figure 4:**
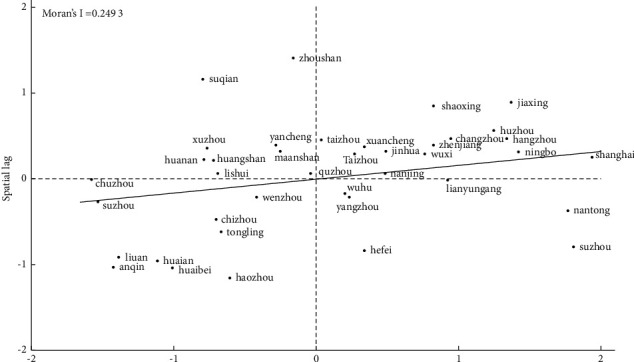
Standardized value of forest development level in 2015.

**Figure 5:**
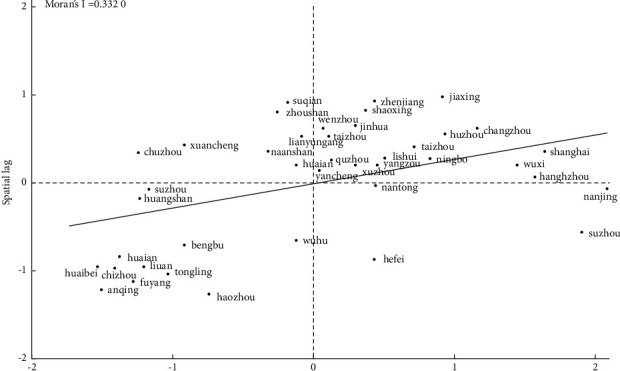
Standardized value of forest development level in 2020.

Figures 4 and 5 show the Local Moran scatter diagram of high-quality forest development of urban agglomerations in the Yangtze River Delta in 2015 and 2020 under the adjacency matrix. It could be found from the figure that Moran's I index is positive and most cities are concentrated in the first and third quadrant, namely, high and low agglomeration areas, indicating that the forest development level of cities in the Yangtze River Delta has a positive spatial correlation.

#### 5.2.4. Forest Carrying Capacity in China (Part of the Area) from 2015 to 2020

**Figure 6:**
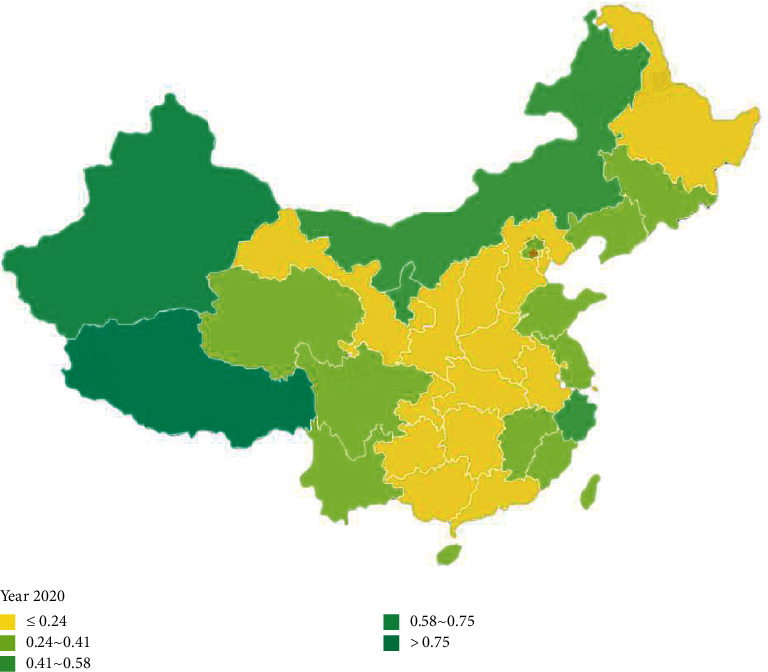
Comprehensive evaluation value of forest environmental carrying capacity (2020 year).

**Figure 7:**
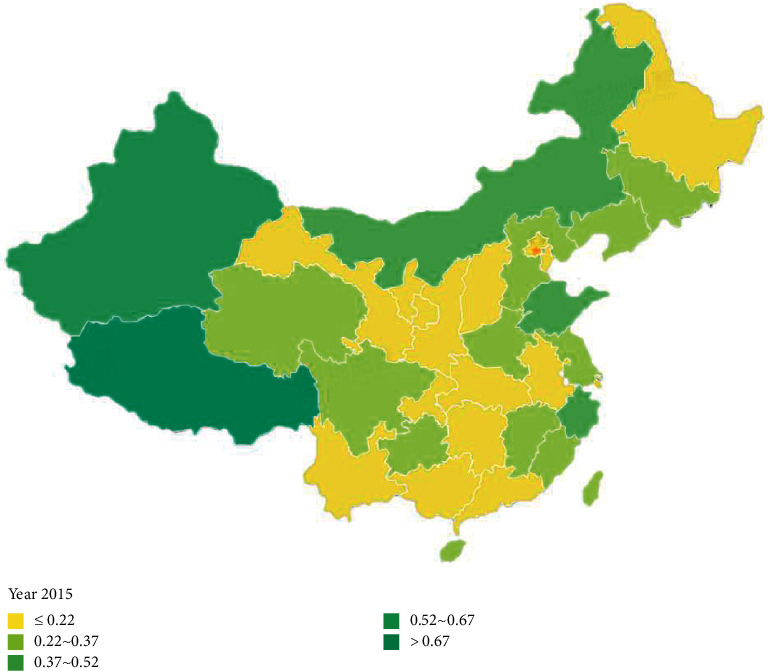
Comprehensive evaluation value of forest environmental carrying capacity (2015 year).

According to Figures 6 and 7, the comprehensive evaluation scores of forest environmental carrying capacity of 34 provinces and cities in China changed from 2015 to 2020. It can be found that: (1) the forest environmental carrying capacity of Tibet, Xinjiang, and Inner Mongolia has always maintained a high level; (2) compared with 2015, the forest environmental carrying capacity of almost all cities in 2020 has been improved, which indicates that the intensity of environmental protection and forest protection in China has been increasing.

## 6. Conclusion

The improvements and contributions of this study mainly focus on the following aspects: (1) a deeply integrated unidirectional bidirectional LSTM considering the correlation of time series is proposed. The bidirectional LSTM network is used to deal with the backward dependence of input data to prevent the omission of some useful information, which is beneficial to the prediction results. (2) In terms of missing data processing, the Mask layer is added to subtly skip the processing of missing data. The results indicate that the proposed method in this paper could predict and evaluate the comprehensive index of forest environmental carrying capacity well. On the basis of this study, further improvement and expansion can be made. A neural network based on graph structure can be added to the model to learn and interpret spatial features.

## Figures and Tables

**Table 1:** The evaluation index system.

Determination of biomass	Sample wood standard	
	Determination of tree layer biomass	Biomass of trunk
		Biomass of branch
		Determination of leaf biomass
		Biomass of root
	Determination of biomass of shrub layer	
	Determination of biomass of herbaceous layer	
	Determination of plant diversity index	
	Classification of forest fire risk levels	

**Table 2:** Forest health grade division criterion.

Health grade	I	II	III	IV	V
Network output value	0	1	2	3	4
Health condition	Healthy	General healthy	Sub-healthy	Unhealthy	Other lands

**Table 3:** Forest health condition of the cities.

Health condition	Small class number
Healthy	7, 37
General healthy	1, 3, 9, 12, 28, 33, 40, 41, 4, 5, 8, 10, 13, 14, 16, 22, 23, 25,
Subhealthy	26, 27, 30, 32, 35, 38, 39
Unhealthy	11, 18, 19, 20, 36
Other lands	21, 29

## Data Availability

Data for this article can be obtained by contacting the corresponding author.
